# Identical Genotype B3 Sequences from Measles Patients in 4 Countries, 2005

**DOI:** 10.3201/eid1211.060635

**Published:** 2006-11

**Authors:** Jennifer Rota, Luis Lowe, Paul Rota, William Bellini, Susan Redd, Gustavo Dayan, Rob van Binnendijk, Susan Hahné, Graham Tipples, Jeannette Macey, Rita Espinoza, Drew Posey, Andrew Plummer, John Bateman, José Gudiño, Edith Cruz-Ramirez, Irma Lopez-Martinez, Luis Anaya-Lopez, Teneg Holy Akwar, Scott Giffin, Verónica Carrión, Ana Maria Bispo de Filippis, Andrea Vicari, Christina Tan, Bruce Wolf, Katherine Wytovich, Peter Borus, Francis Mbugua, Paul Chege, Janeth Kombich, Chantal Akoua-Koffi, Sheilagh Smit, Henry Bukenya, Josephine Bwogi, Frederick Ndhoga Baliraine, Jacques Kremer, Claude Muller, Sabine Santibanez

**Affiliations:** * Centers for Disease Control and Prevention, Atlanta, Georgia, USA;; † National Institute for Public Health and the Environment, Biltoven, the Netherlands;; ‡ Public Health Agency of Canada, Winnipeg, Manitoba, Canada;; § Public Health Agency of Canada, Tunney's Pasture, Ottawa, Ontario, Canada;; ¶ Texas Department of State Health Services, Austin, Texas, USA;; # Instituto de Diagnóstico y Referencia Epidemiológicos, Mexico City, Mexico;; ** Dirección General de Epidemiología de la Secretaria de Salud, Mexico City, Mexico;; †† New Brunswick Department of Health, Fredericton, New Brunswick, Canada;; ‡‡ Pan American Health Organization, Washington, DC, USA;; §§ New Jersey Department of Health and Senior Services, Trenton, New Jersey, USA;; ¶¶ Kenya Medical Research Institute, Nairobi, Kenya;; ## Institut Pasteur, Abidjan, Côte d'Ivoire;; *** National Institute of Communicable Disease, Johannesburg, South Africa;; ††† Uganda Virus Research Institute, Entebbe, Uganda;; ‡‡‡ Laboratoire National de Santé, Luxembourg;; §§§ Robert Koch-Institute, Berlin, Germany

**Keywords:** measles, genotype, epidemiology, surveillance, dispatch

## Abstract

Surveillance of measles virus detected an epidemiologic link between a refugee from Kenya and a Dutch tourist in New Jersey, USA. Identical genotype B3 sequences from patients with contemporaneous cases in the United States, Canada, and Mexico in November and December 2005 indicate that Kenya was likely to have been the common source of virus.

Identification of measles virus genotypes is a valuable tool for epidemiologic investigations and evaluation of control activities in countries that have eliminated indigenous measles. Many of the 23 recognized genotypes of measles are associated with countries or regions with endemic measles (*1*). Measles genotypes in clade B (genotypes B1, B2, B3) are associated with endemic circulation of measles in various countries in sub-Saharan Africa (*2*). The prototype clade B viruses were isolated in 1983 in Cameroon (B1) and in 1984 in Gabon (B2). Hanses et al. (*3*) proposed a new genotype, B3, after characterization of several viruses collected in 1997 and 1998 in Ghana and Nigeria. Sequencing studies of additional viruses from Africa demonstrated that the proposed subdivision of the B3 viruses into subgroups B3.1 and B3.2 was epidemiologically useful for describing 2 distinct clusters of contemporary B3 viruses (*4**,**5*).

Because measles is highly infectious, international travel originating from measles-endemic areas can result in sporadic cases of measles in countries that have eliminated indigenous transmission. International visitors may infect other travelers while moving through transportation hubs or tourist areas; such cases would not be detected unless the traveler sought medical attention or additional cases were detected. Thus, in many of these instances, the source of virus is unknown. We describe the contribution of global surveillance for measles virus genotypes in identifying a common source of virus among contemporaneous cases identified in the United States, Canada, Mexico, and the Netherlands.

## The Study

On November 9, 2005, a 17-year-old man who arrived at the airport in Newark, New Jersey, United States, had symptoms consistent with measles. The man was part of a group of 148 refugees from the Eastleigh community in Nairobi, Kenya, who arrived in the United States from November 3 through 15. Genotype B3 (subgroup B3.1) was identified from virus samples from this patient; the sequence was identical to sequences from measles viruses collected in Nairobi and Machakos, Kenya, in October 2005 (Figure). All but 1 of the 6 viruses collected from Nairobi (Figure, MVi/Nairobi.KEN.xx.05) were from patients from the Eastleigh area of Nairobi, where an outbreak of measles had been reported in the Somali and Ethiopian communities (*6*).

**Figure:**
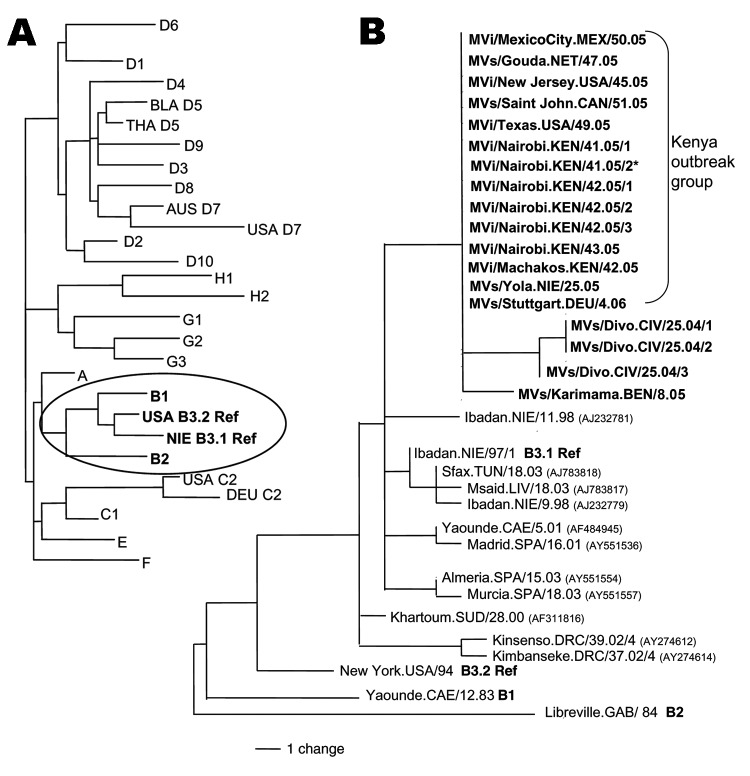
A) Dendrogram showing the relationships among the measles reference strains representing the 23 known measles genotypes (B3 has 2 reference strains). Clade B (circled) is expanded in panel B. B) Midpoint-rooted maximum parsimony tree of nucleoprotein genes (450 nt) of measles viruses from patients in the United States, Mexico, the Netherlands, Canada, and Kenya during 2005 and 2006. The unrooted tree includes sequences from Nigeria in 2005, Germany in 2006, Côte d'Ivoire in 2004, and Benin in 2005 (sequences in bold, this study) as well as selected B3 sequences available from GenBank for comparison. GenBank accession numbers are shown in parentheses. The identical sequences from the "Kenya Outbreak Group" are represented by GenBank accession number DQ888751, MVi/New Jersey.USA/45.05. The GenBank numbers for the sequence from Benin (BEN) and the 3 sequences from Côte d'Ivoire (CIV) are EF031461, EF031458, EF031459, and EF031460. *Collected from the Dagoretti area of Nairobi; the other Nairobi sequences were from cases in the Eastleigh area.

Also in November 2005, a single case of measles was reported in the Netherlands. This patient had visited New York City, returned to the Netherlands on November 15, noted a rash on November 23, and was hospitalized with pneumonia and fever on November 24. The initial investigation focused on potential settings where exposure may have occurred in New York City. The source of infection was traced to an unrecognized exposure to the patient in New Jersey only after analysis of the Netherlands viral sequence demonstrated complete identity with the New Jersey genotype B3 virus. The possibility of an epidemiologic link between the 2 cases led to the discovery that the Dutch visitor had arrived at the Newark airport on November 9 and waited in the arrival area for 1 h, along with the group of refugees from Nairobi.

Subsequently, genotype B3 was identified from patients who had had measles during December 2005 in Texas, Canada, and Mexico. In Texas, during the first 2 weeks of December, 3 cases of measles were reported in members of a family from Houston. The patients had flown directly from Houston to the resort area of Cabo San Lucas, Mexico, where they stayed from November 22 through 27. In Mexico, health authorities reported 5 cases of measles beginning on December 12 among baggage handlers and other airport workers at the Mexico City airport. In New Brunswick, Canada, a patient developed a rash on December 19.

Although the earlier cases in New Jersey and in the Netherlands could be traced directly to the outbreak in Kenya, the sources of the cases in Texas, Canada, and Mexico were unknown. However, the sequences from Texas, Canada, and Mexico were identical to the sequences directly linked to the outbreak in Kenya (Figure). Measles viruses in the same chain of transmission have identical sequences (*7**,**8*), which indicates that the source of the virus for the cases in Texas, Mexico, and Canada was likely to have been the outbreak in Kenya.

Virus transmission may have occurred through contact with international travelers in airports or during transit because epidemiologic investigations did not detect other measles cases in Cabo San Lucas or Texas. The exception to possible air travel–related exposure was the single case that occurred in Canada. This patient had traveled by car, although investigations found no measles cases in the areas visited: Bangor, Maine (December 2); Boston, Massachusetts (December 3–6); and Portsmouth, New Hampshire (December 6). Two refugees from Eastleigh settled in a state visited by the Canadian patient. They entered Massachusetts on November 10, 2005, and by 21 days after arrival, measles had not developed. However, a measles outbreak was detected in southern Germany in January 2006 (*9*), and the viral sequence matched that of the Kenya outbreak virus (Figure; MVs/Stuttgart.DEU/4.06), which indicates that the source of this outbreak was also likely to have been Kenya. Therefore, B3 viruses with identical sequences could have been introduced into Texas, Mexico, or Canada by travelers infected with B3 virus in Europe.

## Conclusions

Although genotype B3 has been the most frequently detected measles genotype in western and central Africa (*4**,**10**–**12*), ours is the first report of the detection of genotype B3 in Kenya. Moreover, the sequence from a virus isolated in Nigeria in June 2005 (Figure; MVs/Yola.NIE/25.05) was identical to the sequences in the Kenya outbreak group. Although a link has not been established between Nigeria and Kenya, a survey of measles genotypes in Kenya in 2002 detected only genotype D4 viruses (*13*). Sequences of viruses isolated during 2004 and 2005 from Côte d'Ivoire and Benin (this study) were included in our analysis (Figure) because these B3 sequences represent closely related viruses from western Africa.

The analysis and dissemination of viral sequences from measles cases led to the identification of an unrecognized epidemiologic link at an airport and linked sporadic cases in 4 countries that do not have endemic measles to an ongoing outbreak in Kenya. Investigators in the field need to collect adequate specimens for virus isolation. Timely communication of sequence data among epidemiologists and microbiologists is critical for identifying possible links among sporadic cases of measles. The potential for rapid transmission of measles during brief encounters with international travelers underscores the importance of global surveillance of measles virus.
